# Taking Familiar Others’ Perspectives to Regulate Our Own Emotion: An Event-Related Potential Study

**DOI:** 10.3389/fpsyg.2019.01419

**Published:** 2019-06-21

**Authors:** Yi Lei, Yajie Wang, Chaolun Wang, Jinxia Wang, Yixue Lou, Hong Li

**Affiliations:** ^1^ Department of Psychology, College of Psychology and Sociology, Shenzhen University, Shenzhen, China; ^2^ Shenzhen Key Laboratory of Affective and Social Cognitive Science, Shenzhen, China; ^3^ Center for Language and Brain, Shenzhen Institute of Neuroscience, Shenzhen, China; ^4^ Department of Psychology, Faculty of Education and Psychology, University of Jyvaskyla, Jyvaskyla, Finland

**Keywords:** perspective-taking, emotion regulation, positive emotion, familiar other, event related potential

## Abstract

Current research on emotion regulation has mainly focused on Gross’s cognitive strategies for regulating negative emotion; however, little attention has been paid to whether social cognitive processes can be used to regulate both positive and negative emotions. We considered perspective-taking as an aspect of social cognition, and investigated whether it would affect one’s own emotional response. The present study used a block paradigm and event-related potential (ERP) technology to explore this question. A 3 (perspective: self vs. pessimistic familiar other vs. optimistic familiar other) × 3 (valence: positive vs. neutral vs. negative) within-group design was employed. Thirty-six college students participated and considered their own or target others’ feelings about pictures with different valences. Results showed that positive emotional responses were more neutral under a pessimistic familiar other perspective, and more positive under an optimistic familiar other perspective, and vice versa for negative emotional responses. In ERP results, compared with a self-perspective, taking familiar others’ perspectives elicited reductions in P3 (370–410 ms) and LPP (400–800 ms) difference waves. These findings suggested that taking a pessimistic or optimistic familiar other perspective affects emotion regulation by changing later processing of emotional information.

## Introduction

Emotion regulation refers to the processes by which individuals influence what emotions they have, when they have them, and how they experience and express these emotions (Gross, 1998a). As an important prerequisite for successful social competence (Marcus, 2016), emotion regulation has received intense interest in emotional psychological research (Campos et al., 2011; Chen, 2016). A large number of studies have focused on cognitive regulatory strategies proposed by Gross (1998b), which can be understood as the cognitive responses to emotion-eliciting events (Aldao and Nolen-Hoeksema, 2010). But as we know, the brain regions involved in cognitive regulatory strategies, especially the lateral prefrontal cortex, do not fully mature until late adolescence (Gogtay et al., 2004), and can be impaired under significant pressure (Raio et al., 2013).

As a fundamental aspect of social cognition (Schwarzkopf et al., 2014), perspective-taking refers to stepping out of one’s own experience and imagining another individual’s emotions, perceptions, and motivations from that person’s perspective (Galinsky et al., 2006). The essential feature is to consider the other’s viewpoint (Kurdek, 1978; Qureshi et al., 2010). This is important in daily social interactions (Ku et al., 2015; Todd et al., 2015), and is helpful for establishing better social relations, reducing stereotypes of others (Ku et al., 2010; Laurent and Myers, 2011), and improving one’s ability to empathize (Batson et al., 2007).

Perspective-taking is ubiquitous in our daily social interactions as well as our emotions, yet we know little about the relation between the two (Bukowski and Samson, 2016). The brain regions activated in perspective-taking primarily involve the medial prefrontal cortex (mPFC; D’Argembeau et al., 2007; Hynes et al., 2006); the mPFC develops earlier than the lateral prefrontal regions. Thus, in this study, we considered whether perspective-taking might be one method for regulating emotions.

To the author’s knowledge, only three studies have documented the relation between perspective-taking and negative emotion. Gilead et al. (2016) analyzed 24 whole-brain fMRI data obtained while participants attempted to predict the affective responses of tough or sensitive others, and found that affective responses were more negative when participants viewed negative images from the perspective of a sensitive vs. tough other. Hence, perspective-taking may cause people to feel less distressed in the face of adversity. Li and Han (2010) showed that when judging painful images, relative to a self-view, taking a different perspective elicited a lower later component, which suggested that perspective-taking modulates later processing of painful pictures. Xia et al. (2014) also showed that a self-perspective elicited an increased amplitude of N200 and LPC than another perspective when judging the shapes of negative images. They thought that perspective-taking inhibits the processing of implicit emotional information. Based on previous research, we proposed that when judging emotional pictures, the influence of perspective-taking on emotion regulation occurs later in the processing of emotion.

Previous studies have only discussed the relation between perspective-taking and negative emotion, yet emotion regulation includes both negative and positive emotions, and dysregulation of positive and negative emotion is thought to be characteristic of some mental disorders (Watson et al., 1995; Kashdan, 2007). As such, we decided to explore both positive and negative emotions by asking participants to judge the emotional valence of positive and negative pictures. According to the results of Gilead et al. (2016) which found that taking a tough other’s perspective may cause people to feel less unpleasant, we hypothesized that when taking pessimistic familiar other perspective, participants’ judgment will be more negative, and the judgment of pictures will be more positive under optimistic familiar other perspective conditions. In other words, a participant’s judgment of negative pictures will be more neutral under optimistic familiar other perspective conditions, and we will find the opposite effect for positive emotional responses.

Second, previous studies always used general, unfamiliar others as perspective targets, and participants were therefore likely to be influenced by stereotypes when taking an other-perspective. Frantz and Janoffbulman (2000) and Vazire and Mehl (2008) indicated that the similarity and intimacy of others would lead to more successful perspective-taking. Accordingly, in this study, we used familiar others as perspective targets, to ensure successful perspective-taking and avoid as much as possible the tendency for stereotypical knowledge and affective responses to interfere with the results (Gillihan and Farah, 2005).

Third, previous studies on perspective-taking have primarily employed two paradigms (Mu and Han, 2013). One is block paradigm in which participants performed different tasks in different blocks of trials, and the other is event-related design in which participants need to shift to a different perspective in each trial. Most existing studies have adopted the block paradigm (Li and Han, 2010; Mu and Han, 2013; Ma et al., 2014; Xia et al., 2014). We used the block paradigm to present the stimuli, a less cognitively demanding task when shifting perspective continuously.

Additionally, there are some published studies (e.g., Donchin and Coles, 1988; Hajcak et al., 2010) suggesting that P3 amplitude is an effective index of allocation of cognitive resources and selective attention. Leng and Zhou (2010) showed that observing feedback on a friend’s performance elicited stronger P300 responses than observing feedback on a stranger’s performance. In other words, if people pay more attention to a cue, the elicited P3 would be higher. Furthermore, it has been argued that the reduction of late positive potential (LPP) can be used as a valid marker of emotion regulation (Hajcak and Nieuwenhuis, 2006; Thiruchselvam et al., 2011; Cauwenberge et al., 2017).

Given these sources of evidence, our study focused on whether taking pessimistic or optimistic familiar other perspectives would influence participants’ judgment of the valence of pictures by using ERP technology; thus, providing evidence for the correlation between perspective-taking and emotion regulation. We proposed that participants’ judgment to the positive (or negative) pictures would be more neutral under the pessimistic (or optimistic) familiar other perspective condition vs. the self-perspective condition, and that lower P3 and LPP difference waves would be observed when taking a familiar other perspective. That is, we hypothesize a familiar other perspective would influence later processing of emotion.

## Method

### Participants

Thirty-six right-handed healthy college students (male 19, female 17, mean age 20.22 years, SD = 1.57, range 17–24 years) from JiangXi Normal University with normal or normal-to-corrected vision participated in this study, each of them indicated that they would not feel obvious discomfort when viewing photos of snakes, scorpions, and so on. Informed consent was obtained in writing from the legal guardians of participants under 18 years of age, alongside written assent from the participants; those aged 18 years and over provided written informed consent themselves. The investigation was approved by the Medicine Ethics Committee of JiangXi Normal University and was conducted in accordance with the Declaration of Helsinki. After the experiment, they were paid 35 Chinese yuan (about US$5) as compensation.

## Materials

### Questionnaires

#### The Optimism and Pessimism Scale

The descriptions of a pessimistic and a optimistic other in the experimental instruction were excerpted from this scale. For example, “he/she always focuses on the bad side of things.” Xu et al. (2010) found that this scale has high reliability and validity, and can be used for Chinese college students.

#### State-Trait Anxiety Inventory

This questionnaire was compiled by Spielberger, and is one of the most frequently used measures of anxiety. The STAI consists of two questionnaires of 20 items each; the first one measures state anxiety, and the other, trait anxiety (Marteau and Bekker, 1992). Zheng and Li (1997) showed that the Chinese version of this inventory is valid for use in China.

#### Beck Depression Inventory-II

This inventory was used to assess the degree of depression (Beck et al., 1996), Yang et al. (2014) indicated that the BDI can be used with Chinese youth. The BDI contains 21 items; for each item, participants choose one of the four descriptive sentences that best describes themselves.

### Affective Stimuli

A total of 540 pictures were taken from the Chinese Affective Picture System (Bai et al., 2005), including 180 positive pictures (valence: 7.07 ± 0.32, arousal: 5.41 ± 0.72), 180 neutral pictures (valence: 5.54 ± 0.28, arousal: 4.12 ± 0.73), and 180 negative pictures (valence: 2.72 ± 0.41, arousal: 5.46 ± 0.76). All positive, neutral, and negative pictures were divided to three blocks, matched for valence and arousal. The pictures were all resized as 6.0 cm × 4.0 cm, and presented randomly using E-prime 2.0.

## Procedure

Before the experiment began, participants read instructions asking them to write the names of one of their pessimistic and one of their optimistic friends, and to rate their familiarity and intimacy with these two friends (1 = extremely familiar or intimate, 9 = extremely unfamiliar or not intimate). Afterward, the experimenter entered the name cues in the program, and then experiment began.

The experiment consisted of a practice session and the formal experiment. The practice session included nine trials in which each condition was randomly presented in each trial. All the stimuli and tasks were the same as in the formal experiment. The formal experiment included three blocks: one block for self-judgment, one block for the pessimistic familiar other task, and another for the optimistic familiar other task. Each block contained 180 trials and each valence of pictures was presented for 60 trials.

**Figure 1:**
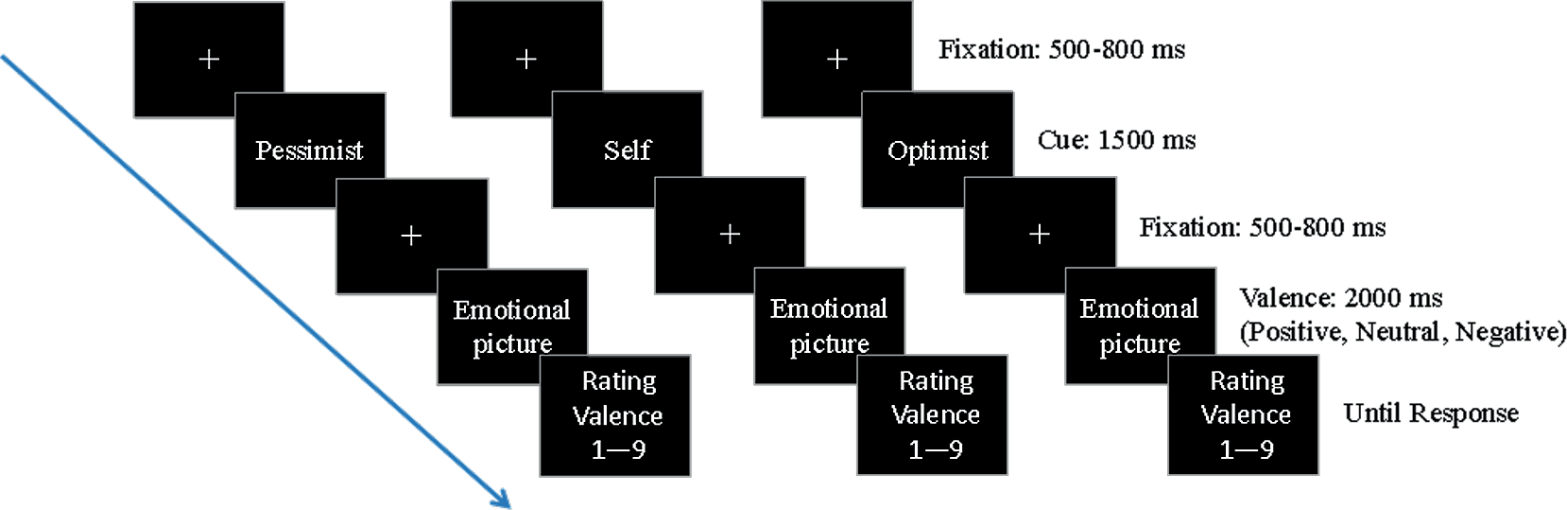
Timeline showing the experimental design of a single trial in each block. The fixation was presented randomly for 500–800 ms, and then the cue name that reminded the participant of the nature of each judgment was shown for 1,500 ms. After 500–800 ms, the fixation was presented again, and the emotional picture was presented for 2,000 ms. Finally, participants judged the emotional picture considering the cue name.

Each trial began with a fixation that was presented randomly for 500–800 ms, and then the cue name that reminded the participant of the nature of each judgment was shown for 1,500 ms. After 500–800 ms, the fixation was presented again, and the emotional picture was presented for 2000 ms. Finally, participants judged the valence of this emotional picture considering the cue name (1 = extremely pleasant, 9 = extremely unpleasant) by showing that “what’s your pleasure level now/consider you are xxx (the cue name), what’s xxx’s pleasure level now, please choose the most suitable option from 1 to 9, where 1 means extremely pleasant and 9 means extremely unpleasant”. After a key pressing response was given, the next trial started. After the task, participants were asked to complete the STAI and BDI (Figure 1).

### Electroencephalographic Recording and Preprocessing

EEG data were recorded from 64 channels using Brain Vision Recorder software (10–20 system), an electrooculogram was recorded below the right eye. Electrode impedance was kept below 10 kΩ and the sampling rate was 500 Hz. All signals were amplified with a bandwidth ranging from 0.05 to 100 Hz.

The data were analyzed by using Brain Vision Analyzer 2.1, and TP9 and TP10 were used as reference electrodes. Using ocular correction, independent component analysis (ICA) was used to correct for artifacts of blinking and eye movement. Data were filtered from 0.1 to 30 HZ, and then epochs were segmented from 200 ms pre-stimulus until 1,000 ms post-stimulus onset, 200 ms pre-stimulus interval was used for baseline correction. Artifact rejection was performed with ±80 μV; calculating the average of the data was the last step.

Based on previous work, P3 was quantified by a mean amplitude measure that used Pz, P3, and P4 in a 350–450 ms time windows (Yuan et al., 2009; Chen et al., 2012; Koivisto et al., 2016); LPP was quantified by a mean amplitude measure that used POz, PO3, and PO4 in a 400–800 ms time window (Babkirk et al., 2014; Van Cauwenberge et al., 2017). Moreover, we decided to analyze difference waves calculated from the amplitude elicited by positive or negative picture judgment minus the amplitude elicited by neutral picture judgment (using the neutral picture judgment condition as a baseline).

### Data Analysis

In the current study, valence rating data were analyzed with a 3 (perspective: self, pessimistic familiar other, optimistic familiar other) × 3 (valence: positive, neutral, negative) repeated measures ANOVA; ERPs (P3 and LPP difference waves for averaged amplitudes) were also analyzed with a 3 × 3 ANOVA. Greenhouse-Geisser and Bonferroni corrections were performed to correct *p* throughout the analysis.

## Results

### Affective Results

The means and standard deviations of the STAI and BDI scores were 44.39 ± 5.09 (state anxiety), 46.50 ± 6.58 (trait anxiety), and 12.86 ± 7.82 (depression). Neither of these measures was significantly correlated with our dependent variable of affect ratings nor did they moderate the effect of perspective (or the interaction of perspective and valence). Thus, they will no longer be discussed.

Paired samples *t* tests were used to compare the familiarity and intimacy scores with participants’ pessimistic and optimistic friends. We found no significant differences between familiarity (*t*_(35)_ = 1.72, *p* > 0.05) and intimacy (*t*_(35)_ = 1.66, *p* > 0.05) between participants’ pessimistic and optimistic friends, and the familiarity and intimacy scores for the same friend had significant correlations (pessimistic other: *r* = 0.75, *p* < 0.05; optimistic other: *r* = 0.90, *p* < 0.05).

We also examined the different between participants’ ratings of pictures and the normative ratings of CAPS using paired samples *t* tests. These tests showed that our participants’ rating of positive, neutral, and negative pictures were more negative than the normative rating of CAPS. The results are shown in Table 1.

**Table 1:** Paired samples *t* tests between participants’ rating of pictures and the normative ratings of CAPS.

	*M*	SD	*t*	*p*
Pos-sPos	−1.57	0.63	−15.03	0.000
Neu-sNeu	−2.20	0.63	−20.97	0.000
Neg-sNeg	−1.30	0.76	−10.26	0.000

*Pos, Neu, and Neg means the normative ratings of positive, neutral, negative pictures in CAPS, respectively; and sPos, sNeu, and sNeg means the rating of positive, neutral, and negative pictures judged by participants, respectively*.

A 3 (perspective: self, pessimistic familiar other, optimistic familiar other) × 3 (valence: positive, neutral, negative) repeated measures ANOVA showed that the main effects of perspective type (*F*_(2,70)_ *=* 28.63, *p* < 0.001, *η*^2^ = 0.45) and valence (*F*_(2,70)_ = 293.46, *p* < 0.001, *η*^2^ = 0.89) were significant, the interaction of perspective type and valence was also significant (*F*_(4,140)_ = 12.34, *p* < 0.001, *η*^2^ = 0.26).

**Figure 2:**
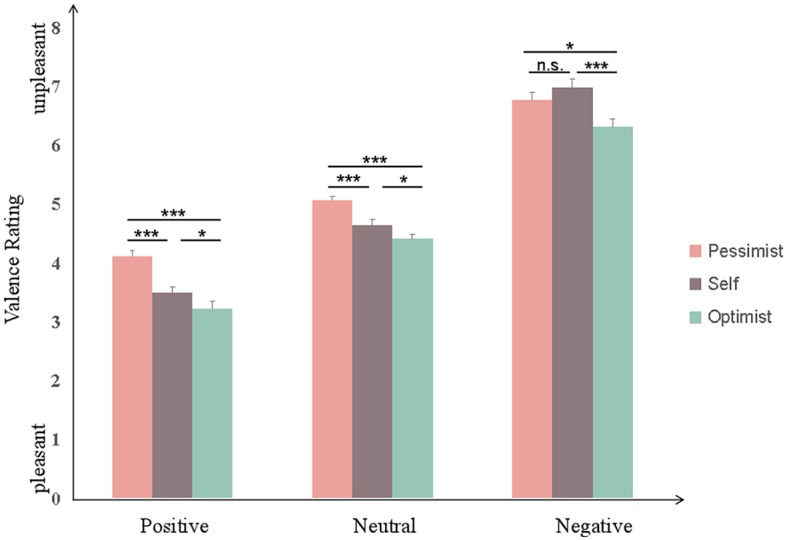
Valence ratings for 36 participants after taking each of the three perspectives. Error bars represent SEM, n.s. means “not significant,” *p < 0.05; ***p < 0.001.

Bonferroni-corrected *post hoc* comparisons showed that when judging positive pictures, ratings of valence were more positive in the pessimistic familiar other perspective condition (*M* = 4.12, SD = 0.63), followed by the self-perspective condition (*M* = 3.50, SD = 0.63), and then the optimistic familiar other perspective condition (*M* = 3.22, SD = 0.76) (all *F* = 19.95, *p* < 0.05). When judging negative pictures, ratings of valence were more negative in the optimistic familiar other perspective condition (*M* = 6.31, SD = 0.77, *F* = 16.03, *p* < 0.05), while the self-perspective condition (*M* = 6.98, SD = 0.80) and pessimistic familiar other perspective condition (*M* = 6.77, SD = 0.83) showed no significant differences (*F* = 16.03, *p* = 0.21). The results are shown in Figure 2.

### Electrophysiological Results

#### P3 Amplitude

**Figure 3:**
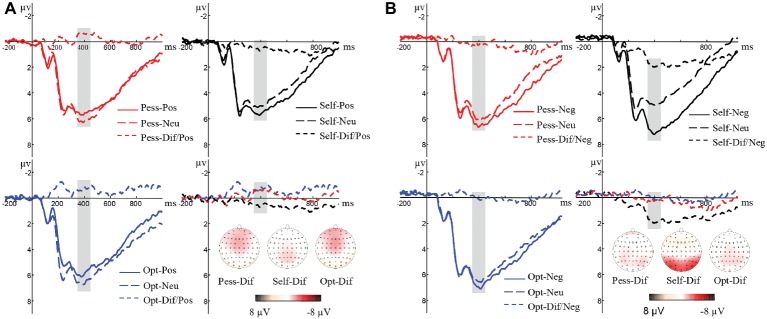
**(A)** Original and difference waves of P3 using average values of Pz, P3, and P4 under positive picture judgment conditions. **(B)** Original and difference waves of P3 using average values of Pz, P3, and P4 under negative picture judgment conditions.

Taking the average peak of three electrodes (Pz, P3, and P4) as P3 amplitude, the ANOVA revealed significant main effects of valence (*F*_(1,35)_ = 5.94, *p* < 0.05, *η*^2^ = 0.15) and perspective (F_(2,70)_ = 31.60, *p* < 0.05, *η*^2^ = 0.47), and a significant interaction of perspective × valence (*F*_(2,70)_ = 24.10, *p* < 0.05, *η*^2^ = 0.41). Bonferroni-corrected *post hoc* comparisons revealed a larger amplitude of the P3 difference wave in the self-condition as compared to the pessimistic and optimistic other perspectives for positive picture judgment (*F*_(2,34)_ = 10.67, *p* < 0.05) and for negative picture judgment (*F*_(2,34)_ = 15.21, *p* < 0.05). The elicited P3 difference wave showed no significant difference between pessimistic and optimistic other perspectives for positive picture judgment (*F*_(2,34)_ = 10.67, *p* = 1.00) or negative picture judgment (*F*_(2,34)_ = 15.21, *p* = 1.00). Original and difference waves of P3 in positive and negative picture judgment conditions are shown in Figures 3A,B.

#### LPP Amplitude

**Figure 4:**
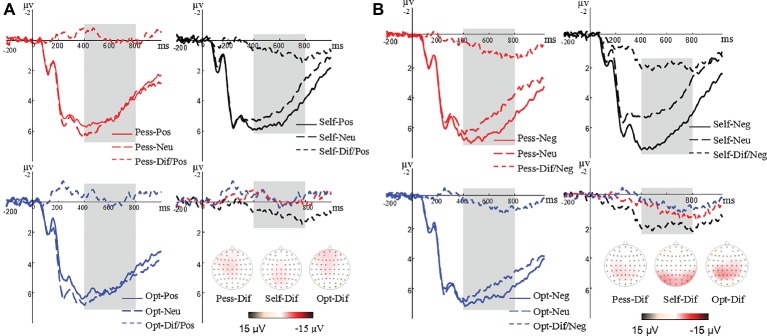
**(A)** Original and difference waves of LPP using average values of POz, PO3, and PO4 under positive picture judgment conditions. **(B)** Original and difference waves of LPP using average values of POz, PO3, and PO4 under negative picture judgment conditions.

Taking the average peak of three electrodes (POz, PO3, and PO4) as LPP amplitude, the ANOVA showed significant main effects of valence (*F*_(1,35)_ = 4.91, *p* < 0.05, *η*^2^ = 0.12) and perspective (*F*_(2,70)_ = 50.55, *p* < 0.05, *η*^2^ = 0.59), and significant interaction of perspective × valence (*F*_(2,70)_ = 17.09, *p* = 0.24, *η*^2^ = 0.33). Bonferroni-corrected *post hoc* comparisons revealed a larger amplitude of LPP difference wave in the self-condition compared to pessimistic and optimistic other-perspectives for positive picture judgment (*F*_(2,34)_ = 17.84, *p* < 0.05) and negative picture judgment (*F*_(2,34)_ = 24.76, *p* < 0.05); the elicited LPP difference wave did not differ significantly between pessimistic and optimistic other-perspectives for positive picture judgment (*F*_(2,34)_ = 17.84, *p* = 0.63) or negative picture judgment (*F*_(2,34)_ = 24.76, *p* = 1.00). Original and difference waves of LPP in positive and negative picture judgment conditions are shown in Figures 4A,B.

## Discussion

This study began with the question of whether or not perspective-taking affects emotion regulation. We hypothesized that participants’ judgment to the positive (or negative) pictures would be more neutral under the pessimistic (or optimistic) familiar other perspective condition vs. the self-perspective condition, and that lower P3 and LPP difference waves would be observed when taking a familiar other perspective. Consistent with our hypothesis, we found that participants’ emotional response was more neutral under a pessimistic (or optimistic) familiar other perspective when judging positive (or negative) pictures, and a reduced amplitude of P3 and LPP difference waves was elicited.

This finding suggested that taking a pessimistic (/optimistic) other perspective to judge positive (/negative) pictures inhibits processing of self-emotion, resulting in partial neutrality of emotional response. When participants judged positive pictures from their optimistic familiar others’ perspective, their own judgment became more positive, which shows that taking an optimistic other perspective to judge positive pictures promotes processing of self-emotion, resulting in partial positivity of emotional response. However, when judging negative pictures, there was no significant interaction between self-perspective and the pessimistic familiar other perspective; two of this conditions’ responses were so negative, that we speculate that participants’ personality traits, specifically their negative judgment tendency, may have subsequently led to this result. Based on previous studies, individuals have attentional and processing sensitivity to negative valence, which would elicit more extreme responses (Luo et al., 2006; Meng et al., 2009). Hence, people with a pessimistic attribution style would judge negative pictures more negatively (Shi and Luo, 2017). Our participants’ negative judgment tendency may have led to higher negative responses when judging negative pictures from their own viewpoint.

The results for the difference waves showed that when judging the valence of pictures, taking a familiar other perspective would elicit lower P3 and LPP difference waves than a self-perspective. P3 is a useful index of attention allocation (Gray et al., 2004; Aggarwal et al., 2010), Thiruchselvam et al. (2012) showed that by altering emotional responses, selective attention plays a fundamental role in emotion regulation, so a higher P3 under the self-view condition means that individuals focused more attention when taking their own view to judge the valence of pictures, which provides evidence for our hypothesis. Moreover, the reduction amplitude of LPP can be observed during down-regulation of positive and negative emotions (Baur et al., 2015); Peng et al. (2013) observed a reduction amplitude of LPP under up-regulation of positive conditions, and the findings of Krompinger et al. (2008) also supported this. In this way, the reduction of LPP when taking a familiar other perspective proved that perspective-taking influences the processing of emotion. Besides, the difference wave of P3 and LPP showed no statistical difference between taking a pessimistic familiar other-view and an optimistic familiar other-view, which shows that the target effect of pessimistic and optimistic familiar other perspectives on emotion are the same. Taken together, the results showed that perspective-taking influences emotion regulation by inhibiting or promoting later processing of emotional information.

Moreover, the results in this study were consistent with those of Gilead et al. (2016), and together provide evidence for the effect of perspective-taking on emotion regulation when taking unknown others’ or familiar others’ viewpoints. The difference of EEG results between this study, and those of Li and Han (2010), and Xia et al. (2014) may show that the mechanism of social emotion regulation is slightly different in the processing of explicit and implicit emotion. By comparing the activation of brain regions in affect labeling (implicit emotion regulation) and reappraisal (explicit emotion regulation) tasks, Payer et al. (2012) found that both explicit and implicit emotion are associated with similar decreases in amygdala activity. Burklund et al. (2014) also found that the two kinds of emotion regulation may utilize overlapping neural processes, although the activation level in the same region differed between the two. We speculate that the activation level may have resulted in the differences in EEG results of explicit and implicit emotion regulation between this study and previous studies, or perhaps the difference between cognitive and social emotion regulation affected the results. This needs to be explored in further research.

Our discovery opens up a series of interesting questions. Why does perspective-taking influence the processing of explicit self-emotion, and what is the mechanism of this effect? Why does perspective-taking affect processing of implicit and explicit self-emotions differently? How can we use this finding in clinical care? Future research should try to answer these questions.

## Limitations and Conclusions

The present study has some limitations that should be acknowledged. First, we know that adolescents’ perspective-taking abilities are still developing (Frick et al., 2014), and the performance of perspective-taking generally declines during adulthood (Ruffman et al., 2008; Zhang et al., 2013). Our research subjects were all college students around the age of 20 years old. As such, our results may not generalize to other groups and ages. Second, we only asked participants to rate valences of pictures, and we did not independently rate the arousal of each emotion in different conditions. Finally, we explored the effect of perspective-taking on antecedent-focused emotion regulation and found it does influence antecedent-focused emotion. Now, we wonder whether perspective-taking would influence response-focused emotion, as it can influence emotions that have already happened. This is interesting, and we can focus on the flexible effect of perspective-taking on emotion regulation.

Despite these limitations, there is little information available in the literature about the relation between perspective-taking and emotion regulation. This study is the first ERP study to explore whether taking pessimistic and optimistic familiar other perspectives would influence our own positive and negative emotions. We found that perspective-taking regulated emotion. This was shown by inhibition of later processing of emotional information, taking pessimistic (or optimistic) familiar other perspectives resulted in partial neutrality of emotional response when judging positive (or negative) pictures. This study sheds new light on emotion regulation from the social view of emotion regulation. Perhaps future studies will confirm that we can regulate our emotions by taking the perspective of a familiar friend.

Future studies can examine the effect of age with larger samples and more comprehensive age ranges. Given the results that when participants judged negative pictures, there was no significant between self-view and pessimistic familiar other-view, we believe personality factors could influence the findings, and future research would do well to investigate how personality style may moderate these relationships. In addition, it is essential that future studies discuss if perspective-taking can be used as a flexible method for regulating emotions.

## Data Availability

The raw data supporting the conclusions of this manuscript will be made available by the authors, without undue reservation, to any qualified researcher.

## Ethics Statement

The investigation was approved by the Medicine Ethics Committee of JiangXi Normal University and was conducted in accordance with the Declaration of Helsinki. Informed consent was obtained in writing from the legal guardians of participants under 18 years of age, alongside written assent from the participants; for aged 18 years and over, written informed consent was obtained from themselves.

## Author Contributions

YLe contributed in designing the study. YW contributed in performing experiments, in the interpretation of data, analysis of data, and writing the manuscript. CW participated in designing the study and analysis of data. JW, YLo, and HL involved in analysis of data. Revision of manuscript was completed by YLe and YW. All authors read and agreed on the final version of the paper.

### Conflict of Interest Statement

The authors declare that the research was conducted in the absence of any commercial or financial relationships that could be construed as a potential conflict of interest.
